# Vision-based egg quality prediction in Pacific bluefin tuna (*Thunnus orientalis*) by deep neural network

**DOI:** 10.1038/s41598-020-80001-0

**Published:** 2021-01-12

**Authors:** Naoto Ienaga, Kentaro Higuchi, Toshinori Takashi, Koichiro Gen, Koji Tsuda, Kei Terayama

**Affiliations:** 1 Graduate School of Science and Technology, Keio University, Hiyoshi, Yokohama, 223-8522 Japan; 2 RIKEN Center for Advanced Intelligence Project (AIP), Nihonbashi, Tokyo, 103-0027 Japan; 3 Tuna Aquaculture Division, Fisheries Technology Institute, Japan Fisheries Research and Education Agency, Nagasaki, 851-2213 Japan; 4 Graduate School of Frontier Sciences, The University of Tokyo, Kashiwa, Chiba 277-8561 Japan; 5 Research and Services Division of Materials Data and Integrated System, National Institute for Materials Science, Tsukuba, Ibaraki 305-0047 Japan; 6 Graduate School of Medical Life Science, Yokohama City University, 1-7-29, Suehiro-cho, Tsurumi-ku, Kanagawa, 230-0045 Japan; 7 Medical Sciences Innovation Hub Program, RIKEN Cluster for Science, Technology and Innovation Hub, Tsurumi-ku, Kanagawa, 230-0045 Japan

**Keywords:** Developmental biology, Marine biology, Machine learning

## Abstract

Closed-cycle aquaculture using hatchery produced seed stocks is vital to the sustainability of endangered species such as Pacific bluefin tuna (*Thunnus orientalis*) because this aquaculture system does not depend on aquaculture seeds collected from the wild. High egg quality promotes efficient aquaculture production by improving hatch rates and subsequent growth and survival of hatched larvae. In this study, we investigate the possibility of a simple, low-cost, and accurate egg quality prediction system based only on photographic images using deep neural networks. We photographed individual eggs immediately after spawning and assessed their qualities, i.e., whether they hatched normally and how many days larvae survived without feeding. The proposed system predicted normally hatching eggs with higher accuracy than human experts. It was also successful in predicting which eggs would produce longer-surviving larvae. We also analyzed the image aspects that contributed to the prediction to discover important egg features. Our results suggest the applicability of deep learning techniques to efficient egg quality prediction, and analysis of early developmental stages of development.

## Introduction

In recent decades, global aquaculture production has grown substantially, contributing an increasing supply of fish for human consumption^1^. The seed stocks of many marine and freshwater species for aquaculture are partially or fully sourced from wild-caught juveniles, which is to the detriment of wild resource management^2^. To preserve wild resources while meeting food demands, a closed-cycle aquaculture system^2^, which does not depend on wild resources, needs to be established. However, hatchery production of aquaculture seeds has not reached a commercial scale for most fish species because of poor and fluctuating larval survival rates^2^. For example, Pacific bluefin tuna (*Thunnus orientalis*; PBT) were reared under aquaculture conditions throughout a complete life cycle in 2002^3^, and rearing techniques for larval culture in indoor facilities are being developed^4–6^. Nevertheless, inconsistent and low survival rates during larval stages still remain significant problems.

One of the major factors limiting the development of efficient seed production is the high variability and unpredictability of egg quality^7^. In most marine fish species, including PBT, the egg quality varies greatly due to, for examples, maternal age and condition factors, the timing of the spawning cycle, overripening processes, genetic factors, and also intrinsic properties of the egg itself^8^. Low-quality eggs generally lead to poor egg survival and hatching success, and gracile larvae with low growth and survival rates and reduced stress resistance^9,10^. Therefore, the development of accurate tools for assessing egg quality before use is required in order to improve the production efficiency of aquaculture hatcheries.

To date, much effort has been directed toward evaluating fish egg quality at early stages based on fertilization success, morphology, biochemical composition, and the transcriptomes of eggs. Although the fertilization rate is a possible indicator of egg quality in salmonids, it is not always correlated with egg quality in marine fish species^9^. Biochemical indicators of egg quality include lipid composition^11^, free amino acids^11,12^, carbohydrates^13^, and enzyme activities related to carbohydrate metabolism^13,14^. Recent reports indicate that the transcriptomic profile of eggs may be correlated with egg quality in rainbow trout (*Oncorhynchus mykiss*^15^), sea bass (*Dicentrarchus labrax*^16^), striped bass (*Morone saxatilis*^17^), and Japanese eel (*Anguilla japonica*^18^). However, egg quality determination by biochemical and transcriptomic methods is disadvantageous for routine hatchery application because the methodologies are time-consuming, and therefore cannot assess the quality of eggs before they are used in larval culture. In contrast, assessment of egg morphology at early embryonic stages could be a simple but useful method of predicting the quality of fish eggs. Several morphological characteristics, including the size, shape, and distribution of lipid vesicles, could be used as predictive criteria to assess hatching success in several marine fish species^19,20^. Moreover, positive relationships between blastomere cleavage patterns and the hatch rates of fish eggs have been observed^21^.

Despite the potential applicability of morphological criteria for rapid egg quality assessment, an accurate and practical egg quality prediction system has not been established due to several obstacles. First, the development of a functional prediction system (e.g., a regression model for egg quality prediction) and its usage require many data sets of egg morphological characteristics to train the system to account for the large variations that generally occur in marine finfish egg morphology^20,22^. Second, a prediction system would only be applicable to a single fish species due to species-specific criteria for egg quality determination^20^. Third, the scoring procedure of morphological characteristics, such as blastomere morphology, is very subjective^21^. Therefore, a simple, accurate, and objective technique for egg quality prediction is required.

Recently, it has been reported that deep neural networks (DNNs), especially convolution neural networks (CNNs), enable the development of high-performance image classification and recognition models with capabilities comparable or superior to those of humans^23–26^. The trained CNN models can also be utilized to visualize and analyze important local features of objects for purposes of image classification and recognition^27^. By applying these techniques to egg quality analysis, it is possible to create a simple, time-efficient, and highly accurate system of egg quality prediction.

In the present study, we successfully used a CNN model to establish a vision-based egg quality prediction and analysis framework for PBT. This framework requires only a photographic egg image for prediction. First, we targeted hatching success as a representative criterion for the egg quality evaluation^8^, and performed a prediction experiment of normally hatching (NH) or not NH prediction. We also conducted an experiment of the survival days (SD) prediction, which is a prediction of whether larval survival will be fewer than or more than four days without feeding. SD is a modified criterion of the survival activity index (SAI) in starvation tolerance test commonly used for evaluation of egg and larvae quality^28–30^. Furthermore, we visualized the aspects of the images that the network focused on to generate a prediction using Grad-CAM^27^. Our results suggest that the proposed framework enables quantified and objective egg quality prediction, which has the potential to improve the production efficiency of aquaculture hatcheries.

## Results

### The proposed framework

**Figure 1:**
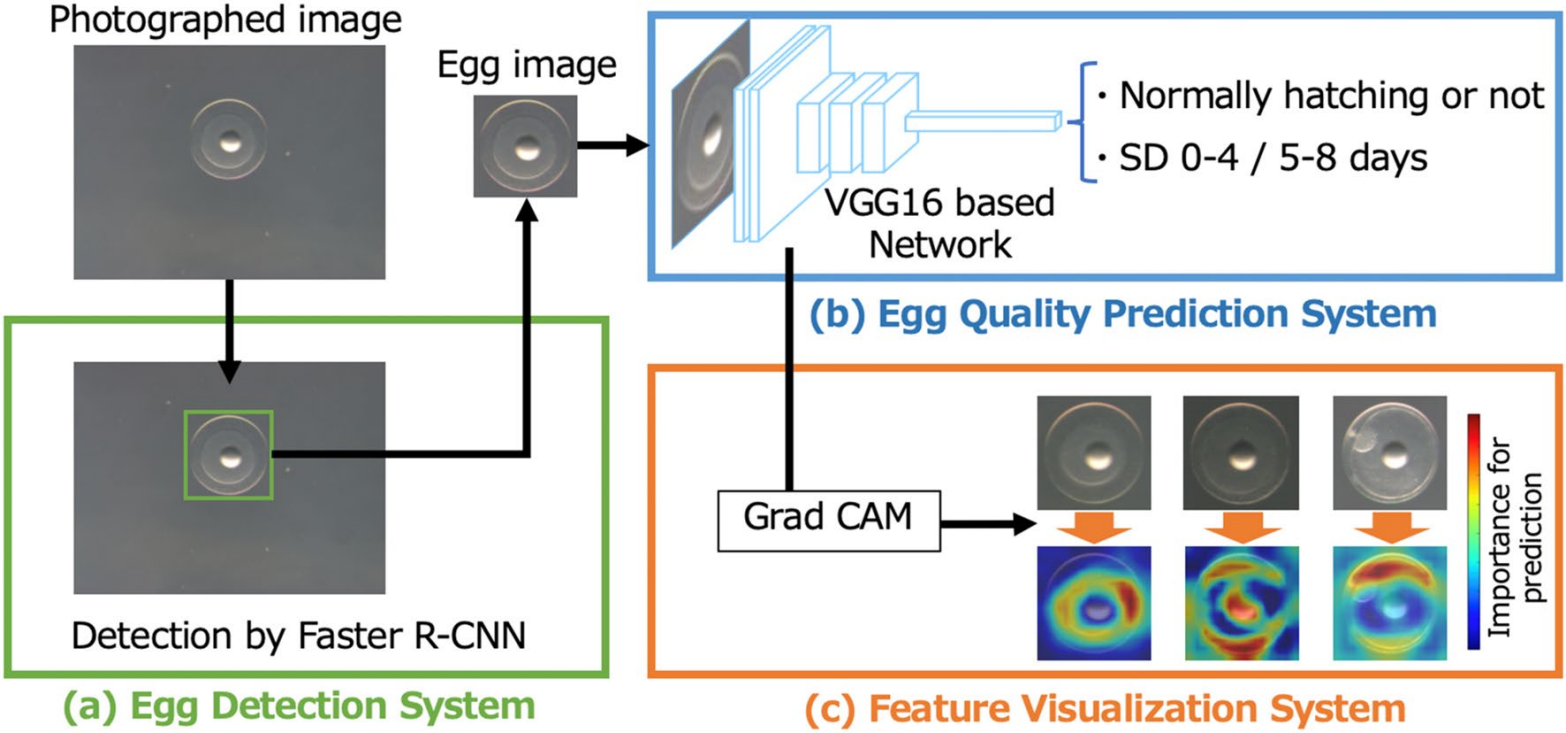
Overview of the proposed framework for egg quality prediction and analysis. (**a**) Only the egg region is extracted from a photographed image by Faster R-CNN. (**b**) By training the VGG16-based network using the egg images, NH and SD predictions are performed. (**c**) The features of the eggs that contributed to the NH prediction were examined by visualization.

Figure 1 shows the overall proposed egg quality prediction and analysis framework. The framework consists of three systems: (a) the egg detection system, (b) the egg quality prediction system, and (c) the feature visualization system. First, an egg is detected by a Faster R-CNN^31^ from the input image (Fig. 1a). The VGG16^32^-based network generates a prediction from the extracted image regarding whether the egg will hatch normally, and whether the SD is within or more than four days (Fig. 1b). Grad-CAM^27^ is used to visualize the aspects of the image most relevant to normal hatching (Fig. 1c). Red or yellow areas contribute greatly to the prediction, while blue areas have little impact on the prediction. See “Materials and methods” for the details of these systems, including the training procedure and detailed parameters of the network. The entire framework enables automatic prediction and analysis of egg quality from only a photographic image.

### Dataset

**Figure 2:**
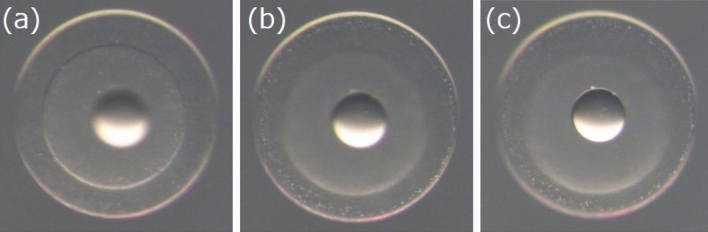
Examples of three types of the input egg images. Images focused on the (**a**) cytoplasm, (**b**) contour of the egg, and (**c**) oil droplet.

290 eggs at the one- to two-cell stage were photographed individually three times, each with a different emphasis on the cytoplasm, contour, or oil droplet of the egg (Fig. 2), resulting in a total of 870 images. The hatching statuses and SDs of the 290 eggs collected from seven spawning events are presented in Tables 1 and 2, respectively. Five hatching statuses were recorded: NH and not NH (this includes malformed hatched [MH], died just after hatching [DH], unfertilized [UF], and unhatched [UH]). See “Materials and methods” for details of the dataset.

**Table 1:** Hatching status of the collected 290 eggs.

NH	Not NH			
	MH	DH	UF	UH
224	6	1	50	9

**Table 2:** SD of the collected 290 eggs.

SD	0	1	2	3	4	5	6	7	8
Number of eggs	60	6	4	1	6	23	132	55	3

### Egg detection

The egg detection system was trained with 144 (the three different emphasis images of 48 eggs) egg images among the 870 egg images. The system was applied to the rest of the egg images (726 images). We visually checked each of the bounding boxes detected by the Faster R-CNN to see if they contained the entire egg, and we found that the system was successful in detecting them in almost all cases except one image (see Supplemental Fig. S1). Thus, the detection accuracy was 0.999 (725/726). In the following processes, we used the egg images extracted from all photographed images by the system. For the exceptional image, we manually extracted its egg image.

### Evaluation metrics

For qualitative evaluation of NH and SD predictions, we calculated the averages of accuracy and F-measure via ten-fold cross-validation, and the area under the curve (AUC) for each of the three types of images. See “Materials and methods” for the details.

### Result of NH prediction

**Figure 3:**
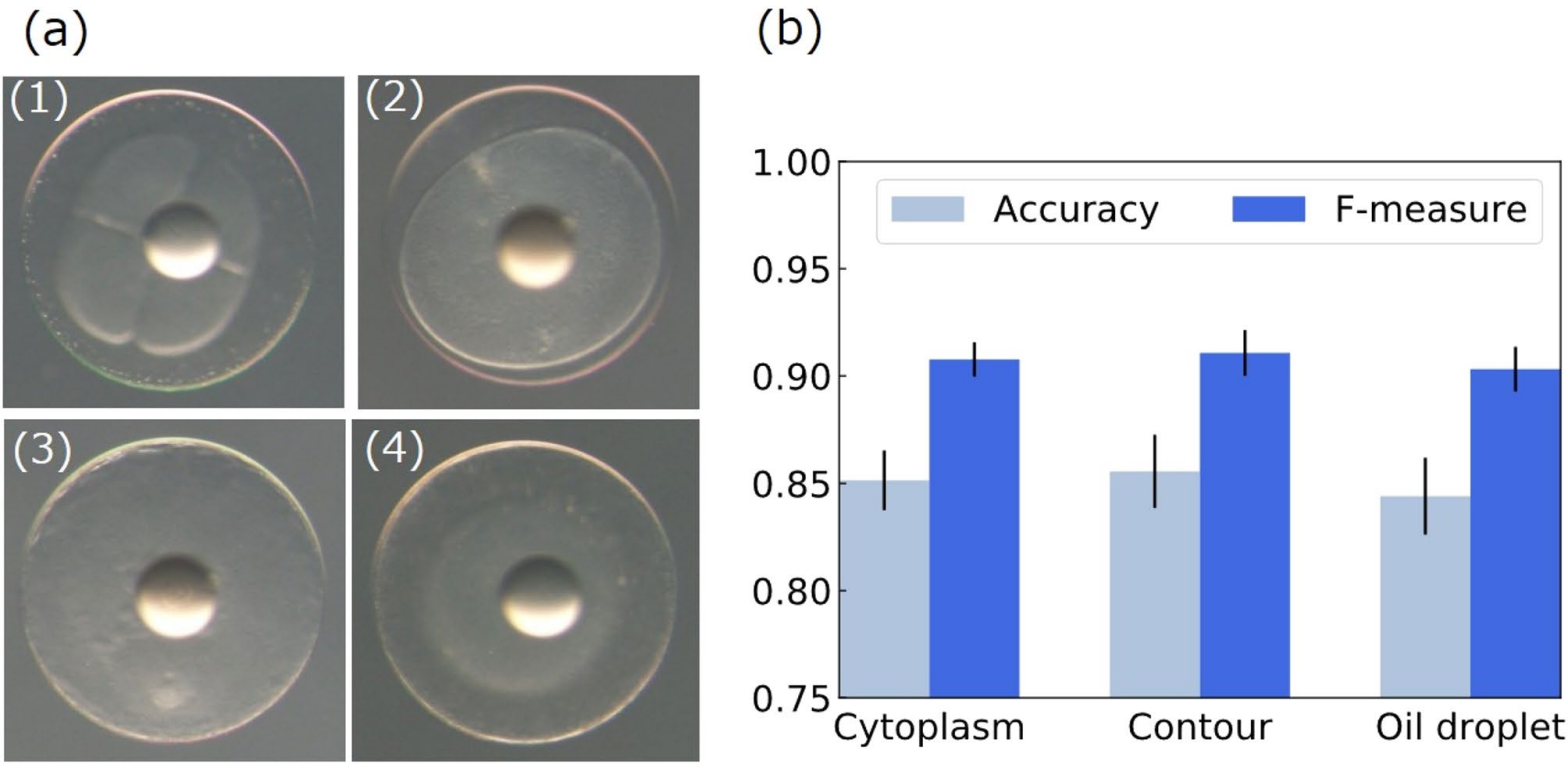
Result of NH prediction. (**a**) (1), (2), (3), and (4) were NH, NH, not NH, and not NH, respectively. The system predicted (1), (2), (3), and (4) as NH, NH, not NH, and NH, respectively. That is, the system successfully predicted for (1), (2), and (3). (**b**) Averages and standard errors of accuracy and F-measure for NH prediction in ten-fold cross-validation.

Three types of the image set (cytoplasm, contour of the egg, and oil droplet) were trained and evaluated separately. The accuracy and F-measure were evaluated via ten-fold cross-validation on 290 images (each image set has 290 images respectively. See “Dataset”). The ratio of the image of each class was made the same for each fold. Figure 3a shows successful and failed examples of prediction by the egg quality prediction system based on contour-focused photographic samples. (1), (2), (3), and (4) were NH, NH, not NH, and not NH, respectively. The system predicted (1), (2), (3), and (4) as NH, NH, not NH, and NH, respectively. That is, the system successfully predicted for (1), (2), and (3). We evaluated NH prediction performances for each focus type (Fig. 3b). The accuracy and F-measure were slightly better for contour images than for cytoplasm or oil droplet images. The accuracy was 0.856 and the F-measure was 0.911 (see Supplemental Tables S1–S6 for confusion matrices and the details regarding accuracy). We also show the receiver operating characteristic (ROC) curve for the contour images in Supplemental Fig. S2; the AUC is 0.812. Prediction accuracy for each spawning event based on the contour-focused egg images is shown in Supplemental Table S14.

### Result of SD prediction

**Figure 4:**
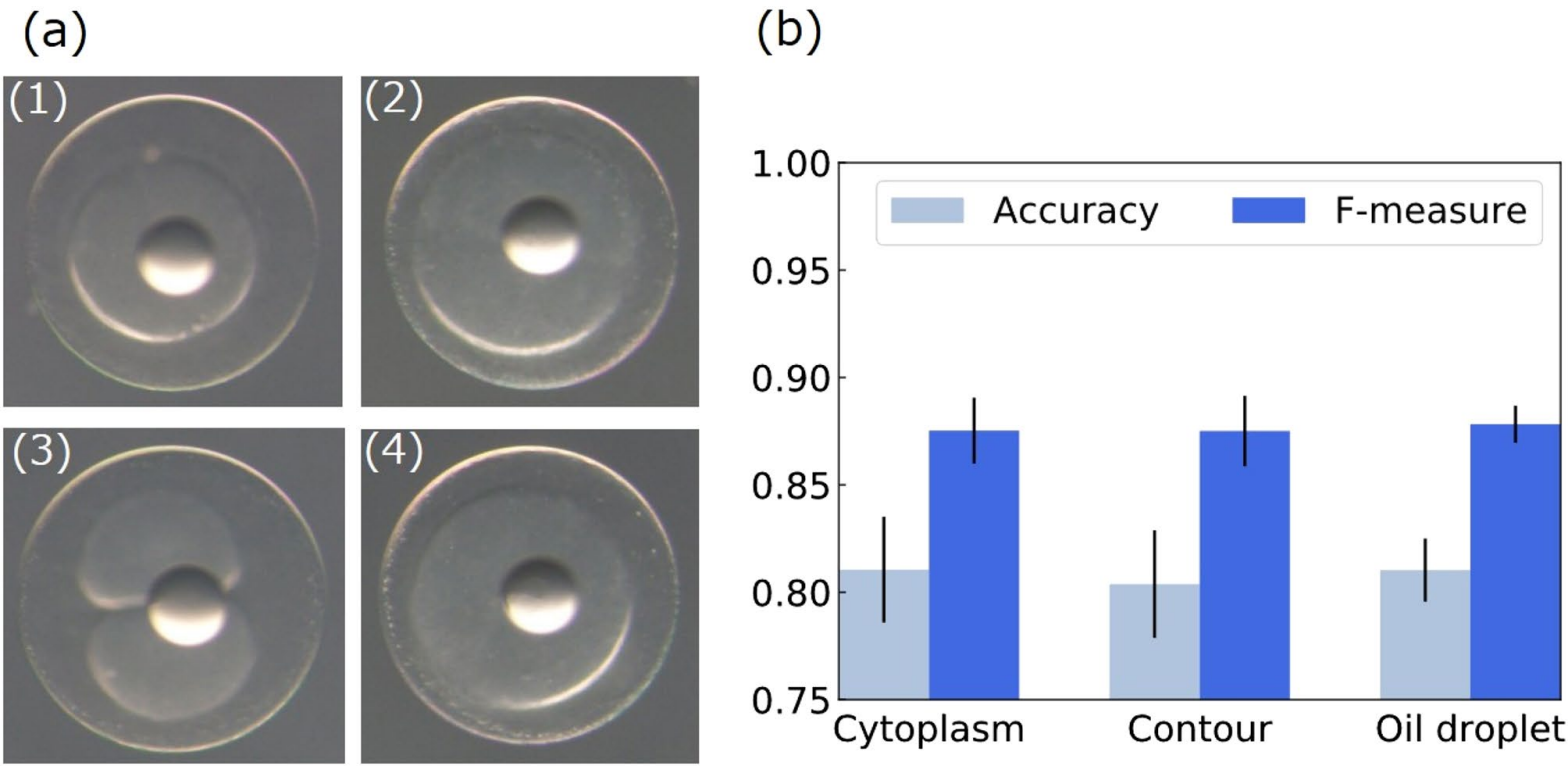
Result of SD prediction. (**a**) The system successfully predicted (1) and (2) but failed in (3) and (4). (1) and (4) were more than four SD; (2) and (3) were NH but within four SD. (**b**) Accuracy averages and standard errors and F-measure for SD prediction in ten-fold cross-validation.

Figure 4a shows successful and failed examples of SD prediction based on contour-focused egg images, all of which were NH eggs. The system successfully predicted the SD outcomes for samples (1) and (2) as more than four and within four, respectively. We evaluated SD prediction performances for each focus type (Fig. 4b). Although SD is difficult to determine from an image alone, the prediction accuracy of the proposed system was 0.804 and the F-measure was 0.875 (see Supplemental Tables S7–S12 for confusion matrices and the details of accuracies). We also show the ROC curve for the contour images in Supplemental Fig. S3; the AUC is 0.789. Prediction accuracy for each spawning event based on the contour-focused egg images is shown in Supplemental Table S14.

### Result of the visualization of image parts that contributed to NH prediction

**Figure 5:**
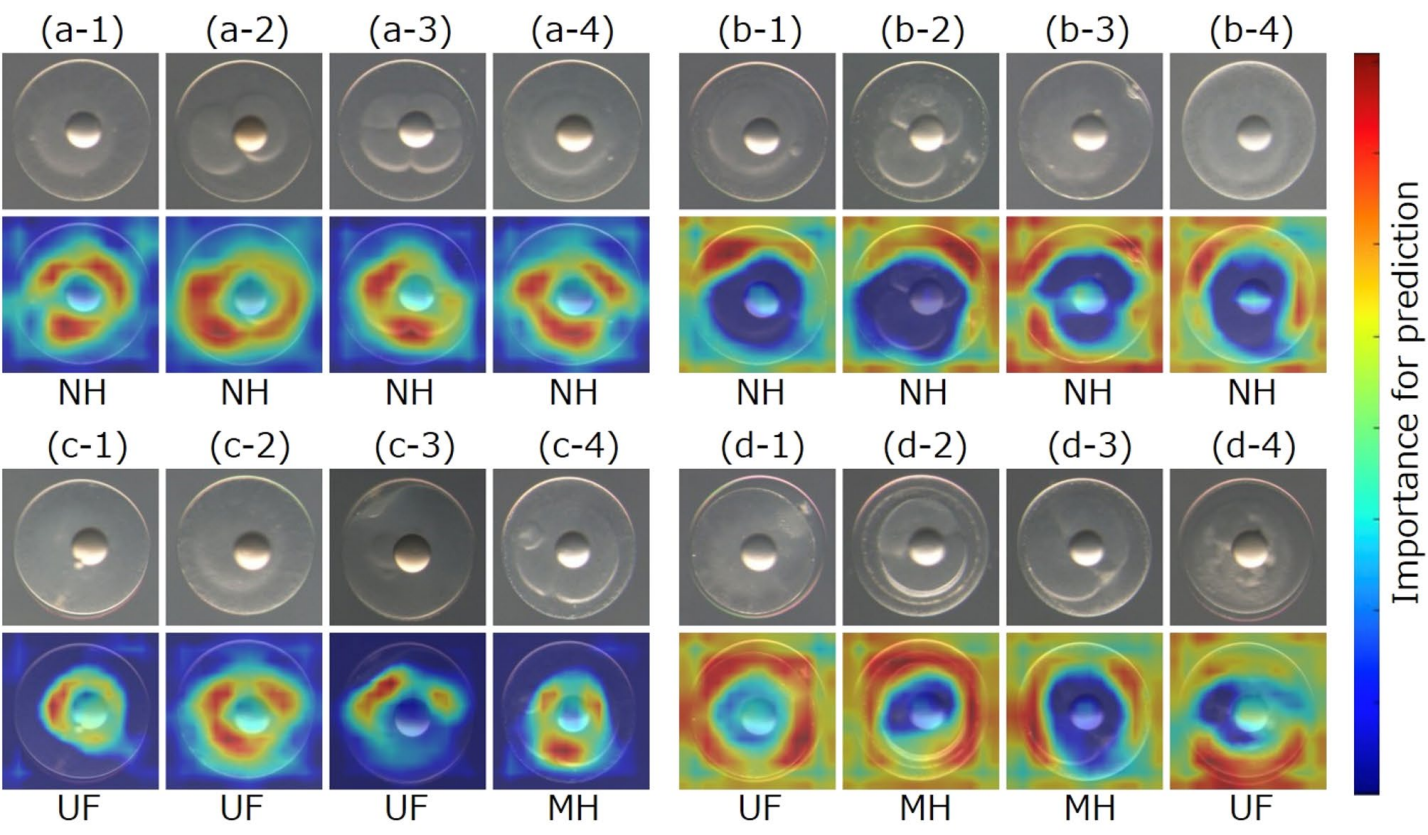
Examples of visualized image aspects that contributed to NH prediction. The images in the first and third rows are the input contour images; those in the second and bottom rows show the parts of the eggs that contributed to the prediction as visualized by Grad-CAM. Images (**a**) and (**c**) were predicted as NH; (**b**) and (**d**) were predicted as not NH. Each acronym below the visualized images indicates the hatching status. That is, examples of true-positive (**a**), false-negative (**b**), false-positive (**c**), and true-negative (**d**), respectively.

Figure 5 shows typical examples of the visualization results based on contour-focused egg images. The network tends to emphasize the cytoplasm when the prediction is NH (Fig. 5a,c), and the chorion when the prediction is not NH (Fig. 5b,d). Causes of not NH were eggshell peeling (Fig. 5d-1, d-2, and d-4) or a dust particle near the chorion (Fig. 5d-3). The network succeeded in capturing these features, which are considered important by human experts. The network correctly predicted Fig. 5d-3 as not NH despite visible cleavage (the egg was MH). Prediction failed in some cases. For example, the above-mentioned abnormal features can be seen in Fig. 5b-2 and 3; however, the eggs were NH. Figure 5c shows examples of anomalous images that the network failed to detect as such.

### Comparison with expert predictions

We compared the prediction performance of the proposed system with that of expert humans, using 50 randomly selected images from the 290 contour images. The ratio of NH and not NH in the subset was matched to the ratio of NH and not NH in the entire dataset. Four experts performed NH prediction. The accuracies for the four experts were 0.80, 0.66, 0.72, and 0.70, respectively. The average accuracy was 0.72 (± 0.051). In comparison, the prediction accuracy of the proposed system was 0.88 (see Supplemental Table S13 for all answers of the four experts and the network).

**Figure 6:**
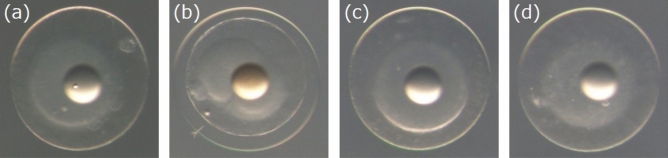
Test image examples to compare the prediction accuracy of experts with the network. (**a**,**b**) Actually NH. The network predicted them correctly while four experts failed. (**c**) Actually not NH. The network and only one expert predicted correctly. (**d**) Actually not NH. The network failed to predict, and three experts predicted correctly.

Figure 6 shows four examples out of the 50 images used for the comparison. The IDs of (a), (b), (c), and (d) are 11, 32, 29, and 21 in Supplemental Table S13, respectively. The eggs in Fig. 6 are difficult to predict. For example, although the cytoplasm in (a) is neatly gathered around the oil droplet, a small bubble that may negatively affect hatching is also present. All experts incorrectly predicted (a) as not NH, whereas the network answered correctly (NH); the same is true of (b). Only one of the experts, as well as the network, correctly predicted (c) as not NH. The network prediction for (d) was incorrect, while three of the experts predicted correctly; there were only two such cases out of 50 images. Further, there were no cases in which all experts answered correctly but the network failed. These results show the potential of the CNN-based approach for the NH prediction.

## Discussion and conclusion

In this study, we proposed a framework by which to predict and analyze whether or not the egg will hatch normally and whether SD is within or more than four days from photographed egg images. Egg quality can be generally defined as the egg’s potential to produce viable fry^8^, whereas good quality eggs for the fish farming industry have been defined as those exhibiting low mortalities at fertilization, hatching, and first feeding^33^. For this reason, NH success is widely accepted as the ultimate measures of egg quality in aquaculture species^34^. Moreover, the previous study has reported that mouth opening of PBT larvae occurs 2–3 days after hatching, and first feeding begins at 3 days after hatching in the rearing conditions^35^, suggesting that more than four SD without feeding is also the dependable criteria for egg quality in the PBT. In fact, mass mortality during first feeding commonly occurs in the hatchery production of bluefin tuna species likely due to low egg quality^36^. Although SD has not been measured as egg quality criteria in bluefin tuna species so far, Gimenez et al.^13^ has analyzed mortality at first feeding (3 days after hatching) in starvation experiment for evaluating egg quality of the common dentex (*Dentex dentex*). Therefore, the proposed framework could predict egg quality in the PBT. In the hatchery production, the precise evaluation of egg quality is one of the most important steps of the mass culture process, allowing the allocation/discarding of eggs or hatched larvae for/from further culture procedures^37^. This study will contribute to improve the production efficiency of aquaculture hatcheries of the PBT.

In this method, the egg region is automatically extracted from the photographed image with remarkable accuracy (99.9%). Such high accuracy stems from the monotonous background of the images, and the fact that only eggs are represented in the images. From these extracted images, the system predicts whether or not the egg will hatch normally and whether SD is within or more than four days. The aspects of the image that contributed to the prediction are visualized. The results of our experiments suggest that the quality of PBT eggs can be predicted with practical accuracy from egg images alone using deep learning techniques. The F-measure, which is an indicator of accuracy, of the NH predictions was 0.911, and that of the SD prediction was 0.875. The accuracy of NH prediction by the proposed method was higher than that by the experts. This result guarantees the high performance of the proposed method.

Most conventional methods of egg quality prediction use quantitatively measurable indicators, such as the size and shape of a lipid vesicle, or lipid droplet distribution. In contrast, our proposed framework based on deep learning techniques demonstrates the system’s ability to evaluate traits related to egg quality that have not been previously considered. In particular, the visualization analysis suggests that both the cytoplasm and the chorion of the egg could be important factors in predicting NH and SD. During normal embryogenesis, the cytoplasm gathers to the egg animal pole, the outline of the cytoplasm becomes clear, and cleavage begins. Therefore, cytoplasm is a reasonable aspect of the egg to emphasize when predicting NH.

From the viewpoint of labor load, it is not realistic for humans to predict individual egg quality based on the above indicators. However, in our proposed framework, the process is fully automated from egg extraction to hatching prediction, so the user only has to take a photograph of the egg. Because our framework enables highly accurate egg quality prediction at a very realistic cost, it is highly practical for use in aquaculture.

As the eggs used in this study were collected from only seven spawning events, they may be sourced from a small number of parent fish. Generally speaking, the more data we have, the better the prediction performance can be expected for machine learning. In the future, we would like to make the proposed framework more robust by using eggs collected from a large number of batches and parent fish. We would expect such diversity, along with a larger quantity of samples, to improve prediction accuracy by reducing bias beyond what we were able to achieve in this study, with its relatively limited quantity of data. The network was able to detect some anomalies that experts did not notice, but overlooked other anomalies that experts did notice (Figs. 5 and 6). These defects can also be solved by increasing the number and variety of images that the network is exposed to.

In the present study, the Grad-CAM analysis shows an emphasis on cytoplasm to predict the hatching success of PBT eggs. Several studies have reported that blastomere morphology is correlated with hatching success in marine fish species, including the Atlantic cod (*Gadus morhua*^38^), Atlantic halibut (*Hippoglossus hippoglossus*^21^), wolffish (*Anarhichas lupus*^21^), and turbot (*Scophthalmus maximus*^9^). In halibut, the five blastomere characteristics at the 8-cell stage (blastomere symmetry, cell size uniformity, cell membrane adjacency, clarity of cell margins, and presence of inclusions) were strongly associated with hatching success^21^. Although human experts were unable to detect differences in blastomere morphology between eggs predicted as NH and not NH in this study, our prediction system might be trained to recognize the key morphological characteristics of blastomeres as criteria for NH predictions. These predictions could be readily validated, as our study design utilizing multi-well culture plates provides a useful means of relating blastomere morphology to the subsequent fate of individual eggs. Based on this potential, it can be said that DNNs can replicate the knowledge of human experts. Moreover, the categorization procedure implemented by the Grad-CAM might be considered more objective in identifying aspects of eggs associated with their quality, as described previously^21,22^. In the future, the proposed framework will allow not only egg quality prediction, but also further elucidation of mechanisms affecting normal and abnormal development in the early developmental stages of various fish species.

## Materials and methods

### Embryo preparation

Three-year-old PBTs were reared in a circular land-based tank (20 m in diameter, 6 m in depth) at Fisheries Technology Institute, Japan Fisheries Research and Education Agency. Eggs were obtained using a net immediately after spontaneous spawning in the tank, and then moved into the laboratory. All animal housing and experiments were conducted in strict accordance with the institutional guidelines for care and use of live fish. The protocols were approved by the institutional committee on the Fisheries Technology Institute, Japan Fisheries Research and Education Agency.

### Photographing PBT eggs and determining egg quality

In this study, a total of 24 spawning events of the PBT were observed between 2 July and 7 August. To investigate morphological characteristics of PBT eggs, we randomly selected 290 eggs out of seven different spawning events and photographed them individually under a stereomicroscope (SZX-7, Olympus, Tokyo, Japan) and digital camera (DP70, Olympus) at 40 × magnification. Each egg was photographed three times, with each photograph differentially focusing on cytoplasm, egg contour, or oil droplet as shown in Fig. 2.

After taking color images, we transferred each egg into a well of a 48-well polystyrene culture plate containing 0.75 ml sterilized seawater and antibiotics (50 µg/ml streptomycin and 50 U/ml penicillin), maintained at 24 °C. At the morula stage (2–3 h after spawning) the eggs were examined under the stereomicroscope to determine fertilization status. After 40 h, NH larvae, MH larvae, DH larvae, UF eggs, and UH eggs were examined. Hatched larvae were subsequently reared in the culture plate without feeding to assess SD.

### Egg detection system

As shown in the upper left quadrant of Fig. 1, the original egg images were 1600 × 1200 pixels, with an egg approximately 450 pixels in diameter near the center (in some images, there was another egg on the edge). Faster R-CNN^31^, which is a convolutional neural network (CNN) method that performs object detection, was utilized to extract only the egg region from the original image. We used Faster R-CNN in a Mask R-CNN implementation^39,40^. An example of the extraction is shown in Fig. 1.

Fine-tuning is effective for a small dataset. This technique enables the network retraining by slowly adjusting the parameters of only part of pre-trained weight while training with another dataset. We used the pre-trained weight MS COCO^41^ (trained on 80k images) for Faster R-CNN. It was fine-tuned using 144 manually extracted egg images over ten epochs; the other parameters were the default values in the original implementation^40^.

Of the regions detected as the egg in the image, only the region closest to the center of the image was predicted as the egg. The detected region was enlarged by a factor of 1.1. The test was performed on all images other than the 144 images used for fine-tuning. We confirmed by visual inspection that extraction failed in only one image (see Supplemental Fig. S1). The failed extraction was manually retried. Images of the egg region were used in subsequent processing.

### Egg quality prediction system

The deep CNN VGG16^32^ is a representative model used for image classification. VGG16 consists of 13 convolutional layers and three fully connected layers. We used the pre-trained weight, trained on the ILSVRC-2012 dataset, which contains 1.3 million color images in 1000 categories. The input image size is resized to 224 × 224 pixels.

The parameters and the details of training the VGG16-based network for the two tasks (NH and SD prediction) are as follows:

- **Types of input images:** Three types of input images were prepared; one focused on cytoplasm, another on egg contour, and one on the oil droplet (Fig. 2). Each type of the image set consisted of 290 images and was used to train and evaluate the model separately (three models were trained).
- **Data augmentation and image normalization:** We employed the data augmentation technique^23,42^ to improve prediction accuracy and prevent overfitting. Vertical flip, horizontal flip, and random rotation with 180° range were randomly performed on the training and validation images. At the same time, z-score standardization was performed for each image (the average pixel value for each image becomes zero while standard deviation/variance becomes one). For test images, only z-score standardization was performed.
- **Data division:** We applied ten-fold stratified cross-validation to evaluate the model’s predictive ability on the two tasks. All input images (290 images) were first divided into ten subsamples (nine training and validation versus one test). The training and validation images were further divided into four more subsamples (three training versus one validation). The final ratio was training:validation:test = 27:9:4. Each dataset was divided while maintaining the ratio between the two classes; for the NH prediction, the classes were NH or not NH, and for the SD prediction, the classes were more than or within four days.
- **Network:** We retrained the last *l* convolutional layers of VGG16 on our dataset. The three fully connected layers of VGG16 were discarded, and replaced with two new fully connected layers. Each of the two fully connected layers contained *u* and two units, respectively (the two tasks are binarily classified) with a dropout rate *d* between the two layers. The activation functions of the two fully connected layers were ReLU and softmax.
- **Training:** The model was trained and evaluated with ten-fold cross-validation using images focused on cytoplasm, contour, or oil droplet (in any case, there were 290 images). The training was stopped when the categorical cross-entropy loss of the validation data did not drop for 10 consecutive epochs. The optimization method was stochastic gradient descent with a learning rate of 1e−4. Each input was weighted according to the reciprocal of the number of each class, that is, the weight of the minority class was one, and the majority class was less than one. The batch size was 10.

Three parameters, *l*, *u*, and *d*, were decided by a grid search in the range of (11, 9, 6, 3), (1024, 2048, 4096), and (0.2, 05), respectively.

### Feature visualization system

Grad-CAM^27^ was used to examine the egg aspects that were important for quality prediction. The aspects of the image that contributed to the prediction were shown in red by Grad-CAM (Fig. 5). The weight of the fold with the highest F-measure was the Grad-CAM input weight.

### Evaluation metrics

True-positive (negative) means that both the actual value and the prediction are positive (negative). False-positive (negative) means that the actual value is negative (positive), but the prediction is positive (negative). Note that positive means NH (more than four days), and negative means not NH (within four days) for NH (SD) prediction. Accuracy and F-measure were calculated as follows:

$$ {\text{Accuracy}} = TP + TN/(TP + TN + FP + FN) $$

$$ {\text{Precision}} = { }TP/(TP + FP) $$

$$ {\text{Recall}} = TP/(TP + FN) $$

$$ {\text{F-measure}} = 2 \cdot {\text{Precision}} \cdot {\text{Recall}}/({\text{Precision}} + {\text{Recall}}) $$

$$ 0 \le {\text{Accuracy,}}\;\;{\text{Precision}},\;\;{\text{Recall}},\;\;{\text{F-measure}} \le 1 $$

where *TP*, *TN*, *FP*, *FN* are true-positive, true-negative, false-positive, and false-negative respectively.

Accuracy is a basic evaluation index indicating what percentage of the predictions is correct. However, if the data are biased, evaluating with accuracy alone is not reliable. Precision is a measure of how many samples are correctly predicted out of samples that are predicted as positive. That is, precision indicates how accurate the positive prediction is. Recall is an indicator of how accurately the system can predict positive samples as such. The applicability of each indicator varies according to the problem in question; however, in most instances, the harmonic mean (F-measure) is used.

Usually, whether the prediction is positive or negative is determined whether the output value of the softmax function is larger or smaller than 0.5. This threshold can be set arbitrarily by the user. For example, if a higher TPR [same definition as Recall) is desirable even though FPR (FP/(FP + TN)] becomes higher, the user can lower the threshold. A model that provides a high TPR while keeping a low FPR at any threshold is a good model. The plot of TPR and FPR for various thresholds is the ROC curve (Supplemental Fig. S2, S3), and the size of the area under the ROC curve is AUC. AUC can also measure the goodness of the model.

## Supplementary Information

Supplementary Information.

## Data Availability

The data that support the findings of this study are available from the corresponding author upon reasonable request.
