# Supplementary material for: Vision-based egg quality prediction in Pacific bluefin tuna (Thunnus orientalis) by deep neural network
Source: Sci Rep. 2021 Jan 12;11:6. doi: 10.1038/s41598-020-80001-0 (PMC7804258; doi:10.1038/s41598-020-80001-0)
Supplement: Supplementary file 1 — Supplementary Information. [file 41598_2020_80001_MOESM1_ESM.docx]

Supplementary Information for

**Vision-based egg quality prediction of Pacific bluefin tuna (*Thunnus orientalis*) by deep neural network**

Naoto Ienaga^1,2†^, Kentaro Higuchi^3†^, Toshinori Takashi^3^, Koichiro Gen^3^, Koji Tsuda^4,5,2^, and Kei Terayama^2,6,7*^

^1^ *Graduate School of Science and Technology, Keio University, Hiyoshi, Yokohama, 223-8522, Japan*^2^ *RIKEN Center for Advanced Intelligence Project (AIP), Nihonbashi, Tokyo, 103-0027, Japan*^3^ *Tuna Aquaculture Division, Fisheries Technology Institute, Japan Fisheries Research and Education Agency, Nagasaki, 851-2213, Japan*^4^ *Graduate School of Frontier Sciences, the University of Tokyo, Kashiwa, Chiba, 277-8561, Japan*^5^ *Research and Services Division of Materials Data and Integrated System, National Institute for Materials Science, Ibaraki, 305-0047, Japan*^6^ *Graduate School of Medical Life Science, Yokohama City University, 1-7-29, Suehiro-cho, Tsurumi-ku, 230-0045 Kanagawa, Japan*

^7^ *Medical Sciences Innovation Hub Program, RIKEN Cluster for Science, Technology and Innovation Hub, Tsurumi-ku, Kanagawa, 230-0045, Japan*

*Corresponding author: terayama@yokohama-cu.ac.jp (K. Terayama)

^†^These authors equally contributed to this work.

**
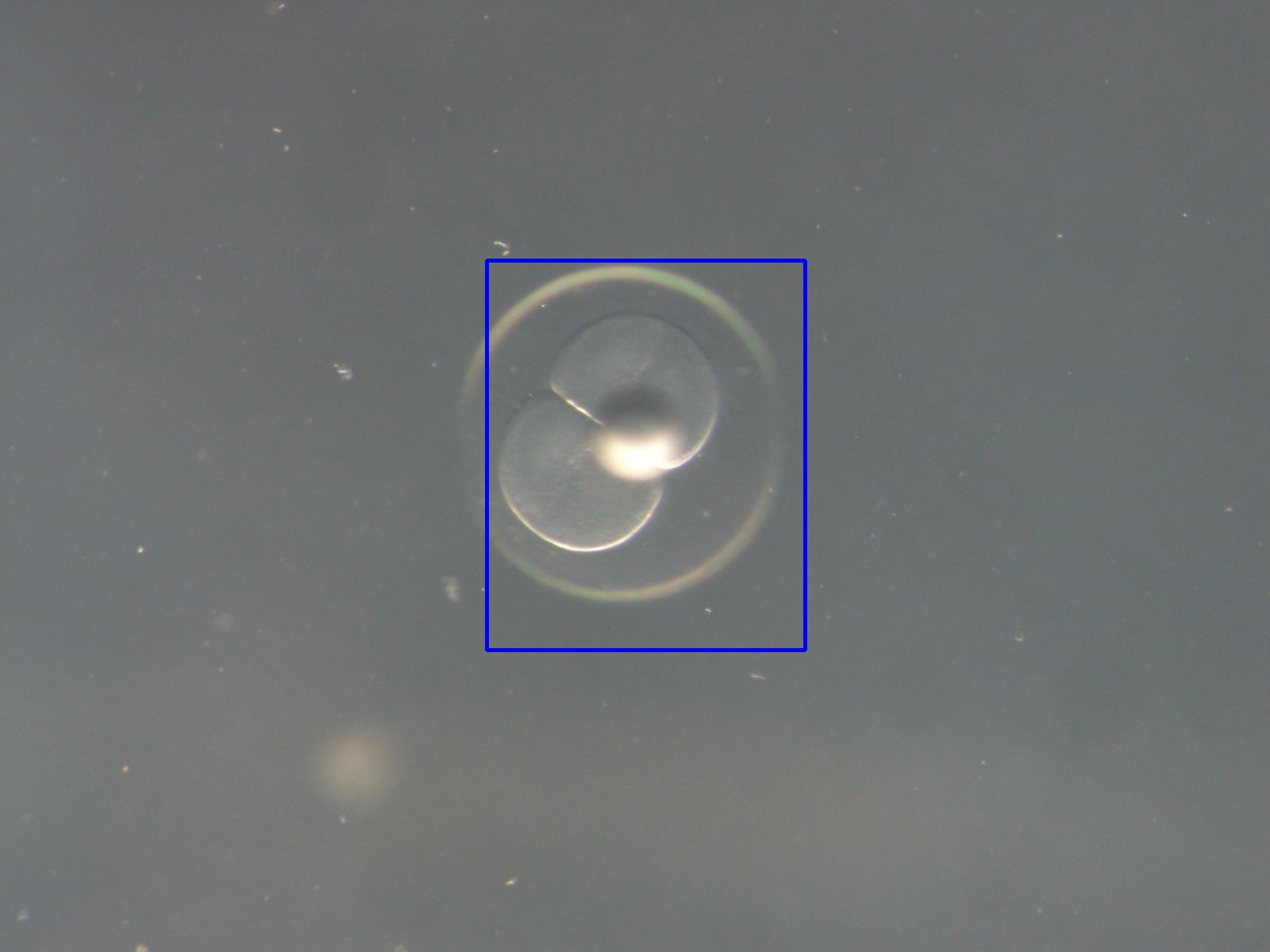
**

**Figure S1. The only one failure case of the egg extraction.**


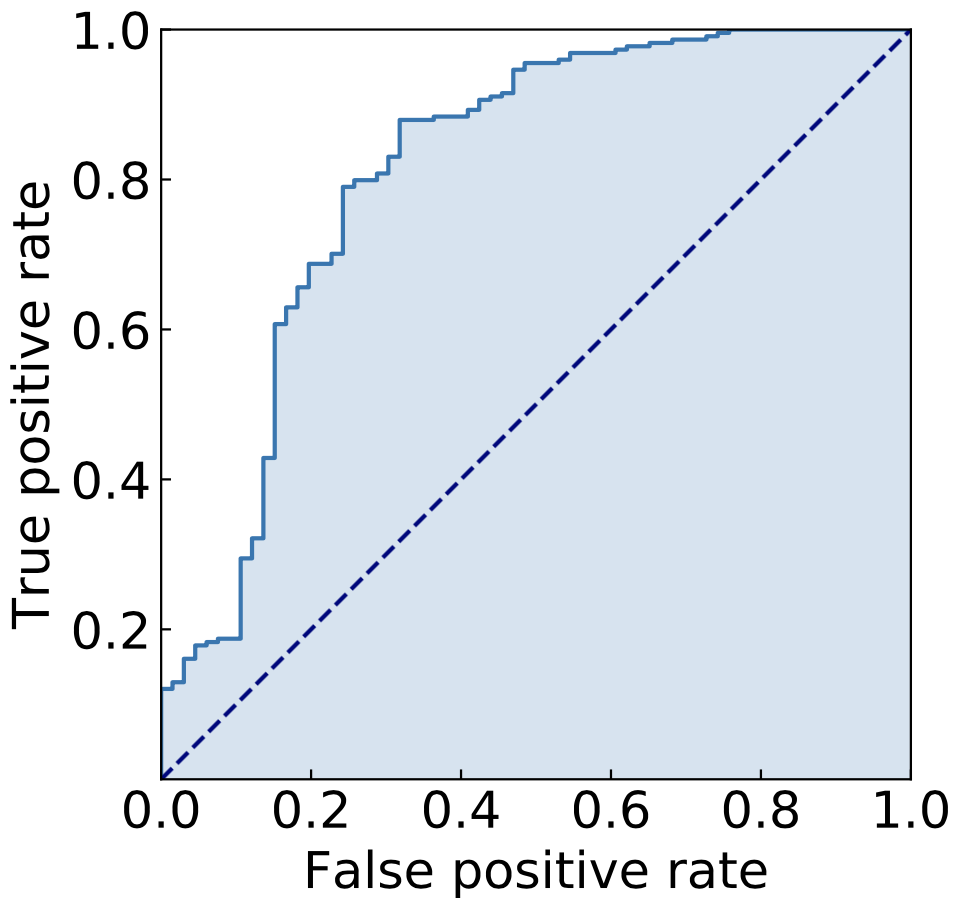


Figure S2. ROC curve for NH prediction using the contour images.


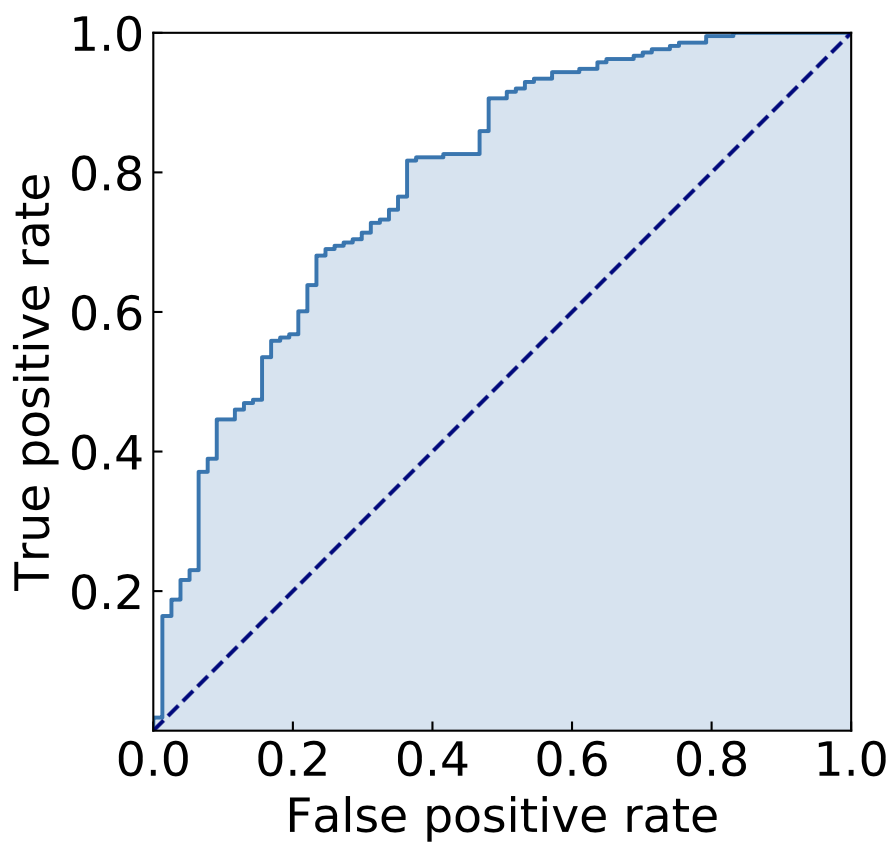


Figure S3. ROC curve for SD prediction using the contour images.

**Table S1. Confusion matrix of NH prediction (cytoplasm).** *l =* 9, *u* = 2048, and *d* = 0.5.

|  | Predicted NH | Predicted not NH |
| --- | --- | --- |
| Actual NH | 211 | 13 |
| Actual not NH | 30 | 36 |

**Table S2. Prediction result of NH prediction (cytoplasm).** *l =* 9, *u* = 2048, and *d* = 0.5.

| Accuracy | Precision | Recall | F-measure |
| --- | --- | --- | --- |
| 0.851 | 0.882 | 0.941 | 0.908 |

**Table S3. Confusion matrix of NH prediction (contour).** *l =* 9, *u* = 2048, and *d* = 0.2.

|  | Predicted NH | Predicted not NH |
| --- | --- | --- |
| Actual NH | 214 | 10 |
| Actual not NH | 32 | 34 |

**Table S4. Prediction result of NH prediction (contour).** *l =* 9, *u* = 2048, and *d* = 0.2.

| Accuracy | Precision | Recall | F-measure |
| --- | --- | --- | --- |
| 0.856 | 0.873 | 0.955 | 0.911 |

**Table S5. Confusion matrix of NH prediction (oil droplet).** *l =* 11, *u* = 4096, and *d* = 0.5.

|  | Predicted NH | Predicted not NH |
| --- | --- | --- |
| Actual NH | 210 | 14 |
| Actual not NH | 31 | 35 |

**Table S6. Prediction result of NH prediction (oil droplet).** *l =* 11, *u* = 4096, and *d* = 0.5.

| Accuracy | Precision | Recall | F-measure |
| --- | --- | --- | --- |
| 0.844 | 0.877 | 0.938 | 0.903 |

**Table S7. Confusion matrix of SD prediction (cytoplasm).** *l =* 9, *u* = 4096, and *d* = 0.5.

|  | Predicted 5-8 SD | Predicted 0-4 SD |
| --- | --- | --- |
| Actual 5-8 SD | 192 | 21 |
| Actual 0-4 SD | 34 | 43 |

**Table S8. Prediction result of SD prediction (cytoplasm).** *l =* 9, *u* = 4096, and *d* = 0.5.

| Accuracy | Precision | Recall | F-measure |
| --- | --- | --- | --- |
| 0.811 | 0.856 | 0.902 | 0.875 |

**Table S9. Confusion matrix of SD prediction (contour).** *l =* 6, *u* = 2048, and *d* = 0.2.

|  | Predicted 5-8 SD | Predicted 0-4 SD |
| --- | --- | --- |
| Actual 5-8 SD | 200 | 13 |
| Actual 0-4 SD | 44 | 33 |

**Table S10. Prediction result of SD prediction (contour).** *l =* 6, *u* = 2048, and *d* = 0.2.

| Accuracy | Precision | Recall | F-measure |
| --- | --- | --- | --- |
| 0.804 | 0.820 | 0.939 | 0.875 |

**Table S11. Confusion matrix of SD prediction (oil droplet).** *l =* 9, *u* = 1024, and *d* = 0.2.

|  | Predicted 5-8 SD | Predicted 0-4 SD |
| --- | --- | --- |
| Actual 5-8 SD | 198 | 15 |
| Actual 0-4 SD | 40 | 37 |

**Table S12. Prediction result of SD prediction (oil droplet).** *l =* 9, *u* = 1024, and *d* = 0.2.

| Accuracy | Precision | Recall | F-measure |
| --- | --- | --- | --- |
| 0.810 | 0.841 | 0.929 | 0.878 |

**Table S13. Table for the comparison with expert predictions using fifty test images for NH prediction.** The checkmarks mean the answer was correct.

| Image ID | Hatching | Expert 1 | Expert 2 | Expert 3 | Expert 4 | Network |
| --- | --- | --- | --- | --- | --- | --- |
| 1 | NH | ✓ | ✓ | ✓ | ✓ | ✓ |
| 2 | NH | ✓ | ✓ | ✓ | ✓ | ✓ |
| 3 | NH | ✓ | ✓ | ✓ | ✓ | ✓ |
| 4 | Not NH |  |  | ✓ | ✓ |  |
| 5 | NH | ✓ | ✓ | ✓ | ✓ | ✓ |
| 6 | NH | ✓ |  | ✓ | ✓ | ✓ |
| 7 | NH | ✓ | ✓ | ✓ |  | ✓ |
| 8 | NH | ✓ | ✓ | ✓ |  | ✓ |
| 9 | Not NH |  | ✓ |  |  |  |
| 10 | NH | ✓ | ✓ | ✓ | ✓ | ✓ |
| 11 | NH |  |  |  |  | ✓ |
| 12 | NH | ✓ | ✓ | ✓ | ✓ | ✓ |
| 13 | NH | ✓ | ✓ | ✓ | ✓ | ✓ |
| 14 | Not NH | ✓ | ✓ | ✓ | ✓ | ✓ |
| 15 | NH | ✓ | ✓ |  | ✓ | ✓ |
| 16 | NH | ✓ |  | ✓ | ✓ | ✓ |
| 17 | NH |  |  | ✓ |  | ✓ |
| 18 | Not NH | ✓ | ✓ |  | ✓ | ✓ |
| 19 | NH | ✓ |  | ✓ | ✓ | ✓ |
| 20 | NH | ✓ |  | ✓ | ✓ | ✓ |
| 21 | Not NH |  | ✓ | ✓ | ✓ |  |
| 22 | NH | ✓ | ✓ | ✓ | ✓ | ✓ |
| 23 | Not NH |  | ✓ |  |  |  |
| 24 | NH | ✓ |  | ✓ | ✓ | ✓ |
| 25 | NH | ✓ | ✓ | ✓ | ✓ | ✓ |
| 26 | NH | ✓ | ✓ | ✓ | ✓ | ✓ |
| 27 | NH | ✓ | ✓ | ✓ | ✓ | ✓ |
| 28 | Not NH |  | ✓ | ✓ | ✓ |  |
| 29 | Not NH |  | ✓ |  |  | ✓ |
| 30 | NH | ✓ | ✓ | ✓ | ✓ | ✓ |
| 31 | NH | ✓ | ✓ | ✓ | ✓ | ✓ |
| 32 | NH |  |  |  |  | ✓ |
| 33 | NH | ✓ | ✓ | ✓ | ✓ | ✓ |
| 34 | NH |  |  |  |  | ✓ |
| 35 | NH | ✓ | ✓ | ✓ | ✓ | ✓ |
| 36 | NH | ✓ | ✓ | ✓ | ✓ | ✓ |
| 37 | NH | ✓ | ✓ | ✓ | ✓ | ✓ |
| 38 | NH | ✓ |  | ✓ |  | ✓ |
| 39 | NH | ✓ | ✓ |  | ✓ | ✓ |
| 40 | Not NH | ✓ | ✓ |  | ✓ | ✓ |
| 41 | NH | ✓ | ✓ | ✓ | ✓ | ✓ |
| 42 | Not NH | ✓ | ✓ |  |  | ✓ |
| 43 | NH | ✓ | ✓ |  |  | ✓ |
| 44 | NH | ✓ |  | ✓ | ✓ | ✓ |
| 45 | NH | ✓ |  | ✓ | ✓ | ✓ |
| 46 | Not NH | ✓ | ✓ |  |  | ✓ |
| 47 | NH | ✓ |  |  |  | ✓ |
| 48 | NH | ✓ |  | ✓ | ✓ | ✓ |
| 49 | NH | ✓ |  | ✓ |  |  |
| 50 | Not NH | ✓ |  | ✓ | ✓ | ✓ |
|  |  | 0.80 | 0.66 | 0.72 | 0.70 | 0.88 |

**Table S14. Image ratio for each class and NH/SD prediction accuracy for each spawning event based on the contour-focused egg images.**

| Spawning event | Ratios of NH and not NH | NH prediction accuracy | Ratios of SD more than four days and within four days | SD prediction accuracy |
| --- | --- | --- | --- | --- |
| 1st | 0.833, 0.167 | 0.667 | 0.833, 0.167 | 0.833 |
| 2nd | 0.638, 0.362 | 0.809 | 0.574, 0.426 | 0.723 |
| 3rd | 0.917, 0.083 | 0.938 | 0.854, 0.146 | 0.875 |
| 4th | 0.542, 0.458 | 0.729 | 0.500, 0.500 | 0.625 |
| 5th | 0.783, 0.217 | 0.848 | 0.761, 0.239 | 0.826 |
| 6th | 0.787, 0.213 | 0.830 | 0.787, 0.213 | 0.830 |
| 7th | 0.958, 0.042 | 1.000 | 0.917, 0.083 | 0.938 |
